# Interventions to improve health and the determinants of health among sex workers in high-income countries: a systematic review

**DOI:** 10.1016/S2468-2667(22)00252-3

**Published:** 2022-11-02

**Authors:** Luke Johnson, Lucy C Potter, Harriet Beeching, Molly Bradbury, Bella Matos, Grace Sumner, Lorna Wills, Kitty Worthing, Robert W Aldridge, Gene Feder, Andrew C Hayward, Neha Pathak, Lucy Platt, Al Story, Binta Sultan, Serena A Luchenski

**Affiliations:** a Department of Primary Care, Population Sciences and Medical Education, University of Southampton, Southampton, UK; b Collaborative Centre for Inclusion Health, Department of Epidemiology and Public Health, University College London, London, UK; c Centre for Public Health Data Science, Institute of Health Informatics, University College London, London, UK; d Centre for Academic Primary Care, Department of Population Health Sciences, Bristol Medical School, University of Bristol, Bristol, UK; e Oxford University Hospitals NHS Foundation Trust, Oxford, UK; f Department of Psychology, The American University of Paris, Paris, France; g Find & Treat, University College London Hospital, London, UK; h Centre for Primary Care and Public Health, Queen Mary University, London, UK; i Guy's and St Thomas' NHS Foundation Trust, London, UK; j Faculty of Public Health and Policy, London School of Hygiene & Tropical Medicine, London, UK

## Abstract

Many sex worker populations face high morbidity and mortality, but data are scarce on interventions to improve their health. We did a systematic review of health and social interventions to improve the health and wider determinants of health among adult sex workers in high-income countries. We searched MEDLINE, Embase, PsycINFO, CINAHL, the Cochrane Library, Web of Science, EthOS, OpenGrey, and Social Care Online, as well as the Global Network of Sex Work Projects and the Sex Work Research Hub for studies published between Jan 1, 2005 and Dec 16, 2021 (PROSPERO CRD42019158674). Quantitative studies reporting disaggregated data for sex workers were included and no comparators were specified. We assessed rigour using the Quality Assessment Tool for Quantitative Studies. We summarised studies using vote counting and a narrative synthesis. 20 studies were included. Most reported findings exclusively for female sex workers (n=17) and street-based sex workers (n=11). Intervention components were divided into education and empowerment (n=14), drug treatment (n=4), sexual and reproductive health care (n=7), other health care (n=5), and welfare (n=5). Interventions affected a range of mental health, physical health, and health behaviour outcomes. Multicomponent interventions and interventions that were focused on education and empowerment were of benefit. Interventions that used peer design and peer delivery were effective. An outreach or drop-in component might be beneficial in some contexts. Sex workers who were new to working in an area faced greater challenges accessing services. Data were scarce for male, transgender, and indoor-based sex workers. Co-designed and co-delivered interventions that are either multicomponent or focus on education and empowerment are likely to be effective. Policy makers and health-care providers should improve access to services for all genders of sex workers and those new to an area. Future research should develop interventions for a greater diversity of sex worker populations and for wider health and social needs.

## Introduction

Sex work spans a wide range of activities, but is defined in this Review as the provision of sexual services in exchange for money or goods. Sex workers are a heterogeneous population—there is extensive variability in the structural, economic, social, and legal context in which they work and in their health and social needs.^1^

Stigma and the hidden—often transient—nature of sex work restrict the availability of accurate data.^1^ There are an estimated 1 million sex workers in the USA and 70 000 in the UK.^2^, ^3^ There are large research gaps in the understanding of their health needs in different settings. Street-based sex workers are highly marginalised and face disproportionate health inequities and harms related to alcohol and drug use, and sometimes HIV and sexually transmitted infections (STIs), hepatitis B, and hepatitis C.^4^, ^5^, ^6^, ^7^, ^8^ Sex workers can encounter high rates of physical, verbal, and sexual violence from intimate partners, perpetrators posing as clients, and the police.^7^, ^9^, ^10^, ^11^, ^12^, ^13^ They frequently have poor mental health, with increased rates of anxiety, depression, loneliness, post-traumatic stress disorder, self-harm, and suicide.^5^, ^7^, ^14^, ^15^, ^16^ There can be severe, complex social needs and structural determinants underlying these health issues, including homelessness or insecure housing, unemployment, adverse childhood experiences, gender and racial inequality, poverty, sex work criminalisation, and the setting of sex work.^5^, ^7^, ^10^, ^14^, ^15^, ^17^, ^18^, ^19^, ^20^, ^21^ However, many sex workers do not face this severe marginalisation or these adverse health outcomes and remain largely unrepresented in academic literature. The legal context in which sex work occurs varies substantially between countries and can either exaggerate or mitigate these harms, with repressive policing practices and criminalisation worsening health outcomes.^22^

Many sex workers face large barriers to accessing health and social care.^23^ There are few specialist services for this community,^24^ and mainstream services are often unaware of sex working and not tailored to sex workers' needs.^17^, ^25^ Sex workers are often unaware of available services,^26^ and might fear legal implications from being identified as a sex worker.^5^ Additionally, past experiences of judgement and stigmatisation while using services could deter them from seeking care again.^5^, ^17^

WHO guidelines state the importance of high-quality, integrated services to meet the health needs of sex workers.^27^ However, there is little published evidence on effective health and social care interventions for sex workers in high-income countries.^28^ There have been three previous systematic reviews, which have focused on psychological interventions for all sex workers,^29^ HIV and STI behaviour change interventions for female sex workers in the USA,^10^ and interventions for illicit drug use in street-working female sex workers.^30^ A comprehensive understanding of interventions tailored to sex workers is needed. This study aimed to systematically review the evidence of interventions used to improve health and the wider determinants of health for all sex worker populations living in high-income countries.

## Methods

We have adhered to the Preferred Reporting Items for Systematic Reviews and Meta-Analyses guidelines.^31^ Our review protocol was registered with PROSPERO in November, 2019 (CRD42019158674). Our team included authors with lived experience, and authors who had worked with and continue to work with sex workers, to ensure the Review's relevance and contextual insight in interpretation of the data.

### Search strategy and selection criteria

We conducted a systematic literature search in six databases (MEDLINE, Embase, PsycINFO, CINAHL, the Cochrane Library, and Web of Science). We used a combination of subject headings and keyword searching related to sex work and health interventions (appendix pp 2–3). Grey literature was also searched using EthOS, OpenGrey, and Social Care Online, the Global Network of Sex Work Projects, the Sex Work Research Hub, and by contacting academic experts and people with lived experience of sex working. Further studies were identified through searching reference lists and citations of included studies. Studies were restricted to those published in English between Jan 1, 2005 and Dec 16, 2021.

### Eligibility criteria

Eligibility was defined using population, intervention, control, and outcomes criteria. The included population were current sex workers, which we defined as people who had exchanged sex for money, drugs, or other goods within the past 12 months. Trafficking and indirect sex work (in which there is no physical contact of any kind with the client) were not included. We included studies with sex workers aged 18 years and older in high-income countries, as defined by The World Bank.^32^ Any intervention with data specifically for sex workers was included. Studies with populations that did not entirely consist of sex workers, and for which—following contact with the authors—disaggregated sex-worker-specific data were not available, were excluded. If the majority of a study population was older than 18 years, and the data was specific to sex workers, the study was still included even if disaggregated data was not available following author contact. Any intervention that studied outcomes related to health or the wider determinants of health (eg, housing and welfare support) was included. Studies of sex work laws were excluded as these were investigated in a systematic review in 2018.^22^ Control groups were not specified a priori.

The review included all quantitative study designs to summarise study effectiveness: randomised controlled trials, quasi-experimental studies (ie, uncontrolled or controlled before-and-after studies), observational studies (ie, cohort, case-control, time series, and cross-sectional), and mixed-methods studies with a quantitative component.

### Data extraction and quality assessment

Titles and abstracts were single-screened for inclusion by one of two reviewers (MB or BM). Remaining articles were double screened at full-text review by two independent reviewers (LCP and BM). Discrepancies were resolved through discussion.

Data extraction was done by one of three reviewers (LJ, LW, or HB) with accuracy checked by a second reviewer (LJ, KW, LW, or HB). Discrepancies were resolved through discussion or decided by a third reviewer (LCP) when they could not be resolved. A spreadsheet was used to extract a standard set of data on study and population characteristics, design, intervention, control, outcome, and results.

Rigour was assessed using the Effective Public Health Practice Project's Quality Assessment Tool for Quantitative Studies,^33^ chosen due to its comprehensive assessment of both observational and experimental studies, and showed reliability and validity.^34^ Criteria assessed include selection bias, study design, confounders, blinding, data collection methods, withdrawals, intervention integrity, and analyses.

### Data synthesis

Due to heterogeneity in method, interventions, and outcomes, we used descriptive vote counting^35^ alongside a narrative synthesis^36^ to summarise findings, following guidance from the Cochrane Handbook for Systematic Reviews of Interventions.^35^

For the narrative synthesis, categories of intervention were developed based on the included papers, and interventions with multiple components were allocated to as many categories as relevant. Intervention components identified were education and empowerment, drug treatment, sexual and reproductive health care, other health care (eg, vaccination, screening, and primary care), and welfare. We summarised the papers in each intervention category according to four main areas: the nature of the interventions, outcomes reported, what was effective, and what was ineffective. We report outcomes as described in the studies but recognise that outcomes relating to cessation or reduction of sex working might not be wanted or important for many sex workers.

To quantitatively analyse results, we used vote counting, which can be used when outcomes are measured heterogeneously between studies.^35^ Vote counting compares the number of studies in which a particular outcome improved with the number of studies in which that outcome did not improve, based only on the direction of effect and therefore with no measure of the magnitude of effect. All studies that measured outcomes before and after an intervention were included. For randomised controlled trials, both the intervention and control groups were included separately if enough information was available. We did this as most controls were well designed interventions that contributed important results to the Review. Intervention categories mirrored the narrative synthesis; the exception to this was that multicomponent interventions were categorised separately both to prevent double counting and because their effectiveness relies on the entirety of the intervention. Outcomes were grouped together into categories. Only outcome categories measured in two or more different interventions were included. If multiple outcomes were reported within one category for a particular intervention, only the primary outcome was used. If no primary outcome was identified and the results were not all in a single direction, the intervention was labelled as having mixed results for that outcome. No intervention had an outcome (or group of outcomes) that deteriorated within an outcome category. We display these data within a harvest plot, which provides a visual summary of the vote counting.^35^ Additionally, we produced a standard binary metric (benefit or mixed results), which we used to calculate a proportion, 95% CI (binomial exact calculation), and p value (binomial probability test) to show the evidence for each intervention category's effectiveness across all outcome measures.

## Results

### Overview

**Figure 1:**
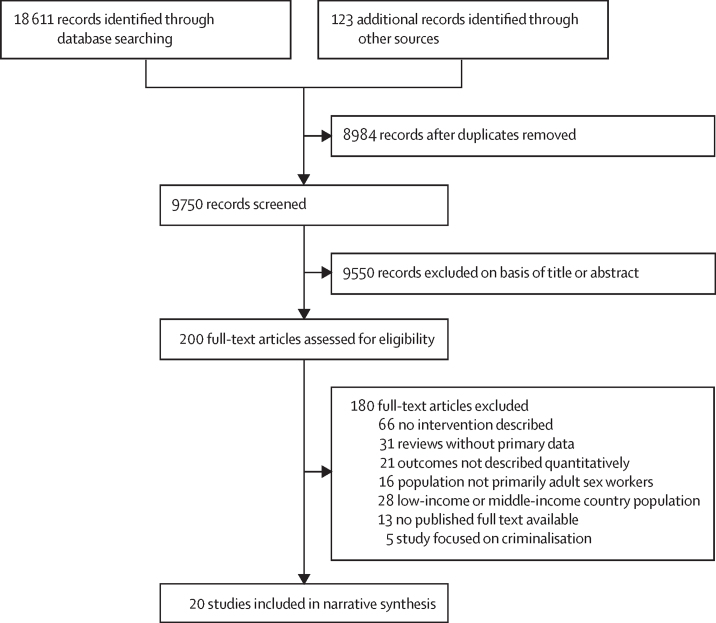
Preferred Reporting Items for Systematic Reviews and Meta-Analyses flow diagram

18 611 studies were identified through database searching and 123 through additional methods. After de-duplication and initial screening, 200 were reviewed in full. 20 studies were included in the final review (figure 1). Summary characteristics and categorisations of included studies are presented in table 1 and table 2. The appendix (p 4) shows a map detailing the number of studies included in each intervention category by country.

**Table 1:** Summary and characteristics of included studies

	**Country**	**Studies (n)**	**Sex worker population**	**Intervention setting or context**	**Intervention**	**Comparison or control**	**Outcomes**	Quality assessment*
**Randomised controlled trials**†								
Wong et al (2019)^37^	Hong Kong	127	Female sex workers aged 18 years and older who had worked in Hong Kong in the past 6 months and who spoke Cantonese or Putonghua	Service centre	Resilience programme consisting of six 1-h sessions designed to improve self-esteem, self-efficacy, and coping skills; it used psychoeducation, cognitive-behavioural strategies, and social learning principles	Standard services provided by NGOs that are specialised in working with female sex workers, which included outreach visits, HIV and STI testing, and social activities	Post-intervention and at follow-up after 3 months, there were significant improvements in resilience, self-esteem, and psychological distress; there was no significant difference in self-efficacy or stress, or STI testing between groups; despite no significant change in condom use in last sexual transaction, there was a significant increase in regularity of condom use in the past week in the intervention group	Moderate
Surratt and Inciardi (2010)^38^	USA	806	Street-based female sex workers who use drugs (heroin, cocaine, or both) who were aged 18–50 years	Unspecified	Sex worker-focused intervention—two 60-min sessions delivered by peers; developed in collaboration with sex workers and focused on how to reduce the HIV risks that sex workers face; also incorporated voluntary testing for HIV, hepatitis B, and hepatitis C	NIDA standard intervention—two 60-min sessions delivered by peers; provided general education on HIV and STI risk reduction and voluntary test for HIV, hepatitis B, and hepatitis C	Follow-up at months 3 and 6; both interventions showed significant decreases in number of days using alcohol or drugs, reduced occasions of sex work while drunk or on drugs, reduced unprotected vaginal and oral sex, and reduced physical and sexual victimisation; sex worker-focused intervention was better at reducing sexual abuse and unprotected oral sex at 6 months; no difference between interventions in drug use reduction, sexual contact reduction, or physical abuse	Moderate
**Cohort studies (one group, pre-intervention and post-intervention)**†								
Cigrang et al (2020)^39^	USA	91	Female sex workers aged 18 years and older with at least one previous arrest for a drug offence and who were currently in jail	Montgomery County Jail, Dayton, OH, USA	Two brief motivational interviews with psychologists, which helped participants identify and prioritise their top concerns for leaving jail, as well as develop plans to address these concerns	NA	Number of arrests significantly reduced from a mean of 3·66 in the 12 months before incarceration to a mean of 1·97 in the 12 months following incarceration (p<0·001); finding safe and affordable housing and controlling use of drugs were the two most commonly cited concerns	Moderate
**Randomised controlled trials**†								
Murnan et al (2018)^40^	USA	68	Female sex workers seeking substance use treatment, with children aged 8–16 years in their care	One intervention group in the participant's home, one intervention group in office setting	Two intervention groups delivered over 6 months: 12 sessions of EBFT in a home or office setting; EBFT targets dysfunctional family interactions that are thought to contribute to the development, maintenance, and resolution of problems	Women's Health Education—12 sessions of individual psychoeducational treatment over 6 months, focusing on topics such as female anatomy, human sexual behaviour, pregnancy and childbirth, and STIs and sexual risk behaviour	Women who received an EBFT intervention reported a greater reduction in drug use and depressive symptoms, and greater improvement in mother–child interactions compared with the control group	Weak
Surratt et al (2014)^41^	USA	562	African American street-based female sex workers who use drugs (cocaine, crack cocaine, heroin, or all three) aged 18–50 years	An office near two of the major sex work areas in Miami, FL, USA	Five sessions of a strengths-based, professional–peer intervention delivered over 8 weeks, professionals were case managers; peers were recovering addicts or former sex workers; peers also remained in contact with the participants throughout the 6-month study, providing ongoing support for service linkage	Five sessions of a strengths-based, professional-only intervention delivered over 8 weeks by a case manager	The inclusion of a peer facilitator provided no additional benefit in reducing HIV risk behaviours or increasing health-service use; however, there were significant changes in both intervention and control groups in some HIV risk behaviours (crack use, and number of sexual partners) and increased use of regular medical care and addiction self-help groups; those who were HIV positive at baseline had a greater reduction in HIV risk behaviour (eg, crack cocaine use, number of sexual partners, and frequency of unprotected vaginal sex) in both groups	Weak
**Cohort analytical studies (two groups, pre-intervention and post-intervention)**†								
Kim et al (2015)^26^	Canada	545 (disaggregated sample size after exclusion of people aged 14–18 years)	Street-based female sex workers aged ≥14 years (including transgender women)	WISH drop-in centre	Women-only, sex-work-specific drop-in service for street-based sex workers, which provided food and drink, showers, clothing, reports of recent client violence (named bad date sheets), HIV prevention resources (eg, condoms and syringes), and referrals to social and health services; also, peer education and support programmes, and outreach nursing care sometimes offered	Female sex workers who did not use the WISH drop-in service	Service use was associated with older age, Indigenous Peoples ancestry, injecting drug use, exchange of sex for drugs, and accessing sexual and reproductive health services; 60% visited WISH during the 3 years of follow-up; the services most frequently accessed were food, make-up, clothing, and primary nurse care	Weak
Deering et al (2011)^42^	Canada	242	Cisgender and transgender women street-based sex workers aged ≥14 years who smoked (not including marijuana) or injected illicit drugs in the last month; authors stated only a small number of participants likely to be aged <18 years	Peer-led mobile outreach programme (the MAP, or the MAP van)	A nightly outreach service staffed by a driver, a support worker, and a peer-support worker; it provides a safe space for women to rest, eat, and drink, and outreach staff distribute reports of recent client violence (named bad date reports); HIV prevention resources distributed (eg, condoms, lubricant, and syringes) and referrals made to health and social support, and drug treatment services	Women within the cohort who did not access the MAP van during the 18-month follow-up	Over the 18-month study period, 42% of reports from sex workers stated use of the van; those using the van were more likely to be working with ten or more clients per week, working in isolated public spaces, and using the WISH drop-in centre service (linked to the van); those who used the programme were more likely to have used inpatient addiction treatment services in the past 6 months; there was no significant relationship between use of the van and accessing outpatient drug treatment; youth aged ≤24 years were significantly less likely to access the van	Weak
Burnette et al (2009)^14^	USA	533	Women exchanging sex for money or drugs in the past 12 months	Outpatient or residential treatment programme	Various drug treatment programmes across 71 facilities in the USA (methadone and non-methadone; residential and outpatient); ancillary services also offered—medical services, mental health services, and psychosocial services	Women not reporting exchanging sex for money or drugs at baseline	12 months after discharge from treatment, sex workers had a lower likelihood of abstinence from drugs and alcohol than non-sex workers using the service; of all women in the programme, there was a reduction of 30% in those reporting sex work, and a reduction of sex work transactions in those who continued sex work; women with a longer duration of treatment and those who received more mental health and psychological services were more likely to have stopped sex work; cessation of sex work was predictive of less frequent drug and alcohol use and fewer mental health symptoms at follow-up	Weak
**Cohort studies (one group, pre-intervention and post-intervention)**†								
Park et al (2020)^43^	USA	103	Female sex workers aged ≥18 years who were using opioids obtained illegally (ie, heroin, fentanyl, prescription opioid pills purchased on the street, or all three)	Various settings (eg, mobile van, study office, fast food restaurants, and home visits)	Training and provision of five fentanyl test strips to test drug samples for fentanyl and related analogues that have increased risk of overdose and death; also provided brief overdose risk assessment, tailored harm reduction advice, and naloxone	NA	1 month later, there were significant reductions in illicit opioid use, injection drug use, benzodiazepine use, and solitary drug use; 84% of women used the strips; of the 48 who had a positive test result, 63% still used their drug as originally intended; others used a range of harm reduction behaviours after positive results	Weak
Decker et al (2017)^44^	USA	60	Street-based and venue-based female sex workers	Mobile van that went to both a street location and a venue known to have a lot of sex trade activity	INSPIRE—a single brief semi-structured discussion with an outreach worker about improving safety and reducing HIV risk, as well as how to access violence support services; supplemented with wallet-sized card summarising information; developed in collaboration with female sex workers	NA	12 weeks after completing the intervention, participants used more safety behaviours, made more use of sexual violence and trafficking support programmes, and were more aware of how to report violence to the police than they were before; they were more likely to engage in condom negotiation and less likely to have sex with clients while using alcohol or drugs than before; there was no change in PTSD or depressive symptoms	Weak
Litchfield et al (2010)^45^	UK	34	Adult female sex workers using heroin	General practioner-led primary care drug treatment service that incorporated a specific clinic targeted at female sex workers	Targeted drug treatment programme—prescribed opioid substitution (typically methadone); sexual health interventions and advice, and key-working and psychosocial support were also available	NA	After 1 year, only 33% of participants were still sex workers, quality of life had improved, and heroin use had reduced (in urine samples, 87% positive for heroin at baseline, 72% at 1 year)	Weak
Ward and Roe-Sepowitz (2009)^9^	USA	29 overall: 11 in residential programme and 18 in prison (incarcerated for non-sex working crimes)	Female sex workers	Two settings—a community-based residential programme and a moderate-security prison	The Esuba programme—a psychoeducational therapy group designed to increase awareness of abuse and violence while teaching anger management and communication skills, and developing social support; one session per week for 12 weeks, each lasting 2 h; sessions facilitated by a doctoral student and clinical social worker	NA	Reduction in trauma scores in both groups, with the prison group having greater change (but they also had higher baseline trauma scores than the residential programme)	Weak
Bowser et al (2008)^46^	USA	189	Female sex workers who use drugs	Programme house	Day programme lasting 12 months, providing meals, HIV risk-reduction education, and one-on-one psychological counselling	NA	By the end of the intervention, there was a significant reduction in polydrug use with alcohol; also significant improvement in housing security and a significant reduction in the number of nights per month spent in jail; no significant change in employment	Weak
Sherman et al (2006)^47^	USA	50	Female sex workers aged 18–45 years who had used heroin or cocaine at least once weekly in the past month	Office in the target neighbourhood (in Baltimore, MD, USA; however, exact neighbourhood unspecified)	Six 2-h sessions that taught HIV risk reduction and the making, marketing, and selling of jewellery; there were opportunities to sell jewellery that was made as well; sessions took place twice per week over a 3-week period	NA	3 months after completing the intervention, there were significant reductions in the number of transactional sex partners, all sex partners, and daily injection and non-injection drug use; women who earned more money through jewellery sales had a significantly reduced number of sex trade partners at follow-up	Weak
**Cross-sectional studies**†								
Stewart et al (2020)^48^	USA	50	First 50 women attending the clinic; 31 (62%) of 50 reported transactional sex for food, shelter, or drugs; however, authors were contacted and strongly suspect that all clients engaged in transactional sex	Outreach clinic at a drop-in centre for people experiencing homelessness	3-h weekly clinic involving an infectious disease physician, a nurse, and a medical social worker; the full-time medical social worker was present on site during each clinic session and on non-clinic days for care coordination; clinic provides primary medical care and harm reduction interventions for drug treatment, including buprenorphine–naloxone, family planning, and treatment of STI, PrEP, and HIV care	NA	Primary reasons for seeking care were skin and soft tissue infection, STI and HIV screening, and urinary tract infection; four (10%) of 39 tested women had unplanned pregnancies; four (10%) of 42 were positive for HIV, of which two were new diagnoses; opioid detected in urine of 31 women—nine initiated buprenorphine–naloxone and three already connected to a treatment programme; 11 (48%) of 23 tested positive for *Trichomonas vaginalis*, five (18%) of 28 for gonorrhoea, five (18%) of 28 for chlamydia, none of 13 for syphilis, and 15 (39%) of 38 for hepatitis C; HIV PrEP prescribed to 17 women	Weak
Baars et al (2009)^49^	Netherlands	259	Female sex workers in brothels, clubs, erotic massage salons, erotic bars, window sex work, and sex work zones	Outreach and clinic	Free, targeted national hepatitis B vaccination programme for sex workers (included men and women—but the study only looked at women and other groups at high risk); also screened for hepatitis B at time of vaccination; community health staff periodically visited various sex worker locations (eg, streets and brothels) and offered vaccines there	NA	205 (79%) participants were aware that they could obtain free hepatitis B vaccination; vaccination uptake—of at least one dose—was 63% (82% of who received it through this programme); those who received the vaccination had worked in an area for longer and were more likely to work behind windows than those who did not receive the vaccination; the most important reason for non-participation was lack of time; of those who started the vaccine course, 74 (79%) of 94 participants received three or more vaccinations, 15 (16%) received two vaccinations, and five (5%) received one vaccination; reasons for not finishing were vacation, changed work location, forgetting, laziness, and did not know; 75% received their first vaccination at an outreach location	Weak
Janssen et al (2009)^50^	Canada	100	Street-based female sex workers aged ≥16 years; authors stated that only a small number of participants aged <18 years were included in this study; disaggregated data could not be obtained	Peer-led mobile outreach programme (the MAP, or the MAP van)	An all-week nightly outreach service staffed by a driver, a support worker, and a peer-support worker; provides a safe space for women to rest, eat, and drink; outreach staff distribute reports of recent client violence (named bad date reports); HIV prevention resources distributed (eg, condoms, lubricant, and syringes) and referrals made to health and social support, and drug treatment services.	Street sex workers not accessing MAP	94% of MAP users said the van made them feel safer—16% recalled a time it prevented physical assault, 10% recalled a time it prevented sexual assault	Weak
Sturrock et al (2007)^51^	Australia	71	Male and female sex workers in brothels	Outreach clinics conducted at brothels	Two nurses and one sexual health educator conducted weekly clinics for 4 weeks, three or four times per year	NA	One (2%) of 63 had chlamydia, none of 51 had syphilis, four (33%) of 12 had hepatitis A, 34 (55%) of 62 had hepatitis B, three (9%) of 34 had hepatitis C; of those with hepatitis C, two were new cases—both reported injection drug use and were referred to general practitioners; 42 (62%) of 68 returned for their results; seven (10%) of 68 received treatment; two sex workers received post-coital contraception; 68 (96%) of 71 were female sex workers	Weak
Wong (2007)^52^	Hong Kong	245	Street-based female sex workers	Clinic—outreach workers invited female sex workers	Twice per month clinic providing cervical smear testing; follow-up explanation of results accompanied by a letter for a gynaecologist	NA	236 cervical smears done, 29 (12%) smears were atypical; nine (31%) of the 29 were referred for further management, and 13 (45%) were uncontactable	Weak
Lomax et al (2006)^53^	UK	24	Street-based sex workers	Genitourinary medicine clinic	After a local syphilis outbreak, sex workers were invited to a clinic for STI and HIV screening; transportation was provided to and from the clinic	NA	24 women seen; 15 cases of syphilis were diagnosed in 14 women (one was a case of reinfection); only four were given intramuscular benzathine benzylpenicillin (treatment of choice), the rest declined and were given suboptimal oral treatment; there was one new HIV diagnosis and several other STIs also diagnosed	Weak

EBFT=ecologically based family therapy. MAP=Mobile Access Project. NA=Not applicable. NGO=non-governmental organisation. NIDA=National Institute on Drug Abuse. PrEP=pre-exposure prophylaxis. PTSD=post-traumatic stress disorder. STI=sexually transmitted infection. WISH=Women's Information Safe Haven.

* Subcategorised by study design hierarchy.

† Study categorisations used are derived from the Effective Public Health Practice Project's Quality Assessment Tool for Quantitative Studies.^33^

**Table 2:** Characteristics of included studies by category

		**Studies (n=20)**
**Country**		
USA^9^, ^14^, ^38^, ^39^, ^40^, ^41^, ^43^, ^44^, ^46^, ^47^, ^48^		11 (55%)
Canada^26^, ^42^, ^50^		3 (15%)
UK^45^, ^53^		2 (10%)
Hong Kong^37^, ^52^		2 (10%)
Australia^51^		1 (5%)
Netherlands^49^		1 (5%)
Sex work legal context in intervention setting at time of study*		
Full criminalisation of sex working^9^, ^14^, ^38^, ^39^, ^40^, ^41^, ^43^, ^44^, ^46^, ^47^, ^48^		11 (55%)
Partial criminalisation of sex working^26^, ^37^, ^42^, ^45^, ^50^, ^52^, ^53^		7 (35%)
Criminalisation of the purchase of sex		0
Regulation of sex working^49^, ^51^		2 (10%)
Full decriminalisation		0
**Sex worker's sex, gender, or both**†		
Female^9^, ^14^, ^26^, ^37^, ^38^, ^39^, ^40^, ^41^, ^42^, ^43^, ^44^, ^45^, ^46^, ^47^, ^48^, ^49^, ^50^, ^51^, ^52^, ^53^		20 (100%)
Male^51^		1 (5%)
Transgender women^26^, ^42^		2 (10%)
**Location of sex work**		
Street based^26^, ^38^, ^41^, ^42^, ^43^, ^45^, ^46^, ^47^, ^50^, ^52^, ^53^		11 (55%)
Brothel or indoor based^51^		1 (5%)
Street based and indoor based^44^, ^49^		2 (10%)
Unclear where sex work takes place^9^, ^14^, ^37^, ^39^, ^40^, ^48^		6 (30%)
**Study design**‡		
Randomised controlled trial^37^, ^38^, ^40^, ^41^		4 (20%)
Cohort analytic (two groups, pre-intervention and post-intervention)^14^, ^26^, ^42^		3 (15%)
Cohort (one group, pre-intervention and post-intervention)^9^, ^39^, ^43^, ^44^, ^45^, ^46^, ^47^		7 (35%)
Cross-sectional study^48^, ^49^, ^50^, ^51^, ^52^, ^53^		6 (30%)
**Quality rating**		
Strong		0
Moderate^37^, ^38^, ^39^		3 (15%)
Weak^9^, ^14^, ^26^, ^40^, ^41^, ^42^, ^43^, ^44^, ^45^, ^46^, ^47^, ^48^, ^49^, ^50^, ^51^, ^52^, ^53^		17 (85%)
**Intervention setting**		
Outreach locations^40^, ^42^, ^43^, ^44^, ^49^, ^50^, ^51^		7§ (35%)
Static site^9^, ^14^, ^26^, ^37^, ^38^, ^39^, ^40^, ^41^, ^45^, ^46^, ^47^, ^48^, ^52^, ^53^		14§ (70%)
**Single component interventions**		
Overall^9^, ^14^, ^37^, ^38^, ^39^, ^40^, ^41^, ^43^, ^44^, ^49^, ^51^, ^53^		12 (60%)
	Education and empowerment^37^, ^38^, ^39^, ^40^, ^41^, ^44^	6
	Drug treatment^14^, ^43^	2
	Sexual and reproductive health care^51^, ^53^	2
	Other health care^9^, ^49^	2
**Multicomponent interventions**†		
Overall^26^, ^42^, ^45^, ^46^, ^47^, ^48^, ^50^, ^52^		8 (40%)
	Education and empowerment^26^, ^42^, ^45^, ^46^, ^47^, ^48^, ^50^, ^52^	7
	Drug treatment^45^, ^48^	2
	Sexual and reproductive health care^26^, ^42^, ^48^, ^50^, ^52^	5
	Other health care^26^, ^48^, ^52^	3
	Welfare^26^, ^42^, ^46^, ^47^, ^50^	5
**Peer involvement**		
Developed with peer workers^44^		1 (5%)
Developed and delivered with peer workers^26^, ^38^, ^41^, ^42^, ^50^		5 (25%)
No peer involvement reported^9^, ^14^, ^37^, ^39^, ^40^, ^43^, ^45^, ^46^, ^47^, ^48^, ^49^, ^51^, ^52^, ^53^		14 (70%)
**Outcomes measured**†		
Drug use and drug harm reduction^38^, ^41^, ^43^, ^45^, ^46^, ^47^, ^50^		7 (35%)
Sexual risk behaviours^14^, ^37^, ^38^, ^41^, ^44^, ^45^, ^47^		7 (35%)
Sex worker safety^38^, ^44^, ^50^		3 (15%)
Mental health and wellbeing^9^, ^37^, ^40^, ^44^, ^45^		5 (25%)
Criminal activity^39^, ^46^		2 (10%)
Outcomes related to wider determinants^46^		1 (5%)
Awareness of health-care and support services^44^		1 (5%)
Use of other health-care and support services^37^, ^41^, ^43^		3 (15%)
Sexually transmitted infection treatment^48^, ^51^, ^53^		3 (15%)
Other health-care outcomes^26^, ^38^, ^41^, ^42^, ^48^, ^49^, ^50^, ^51^, ^52^		6 (30%)

Data are presented as n or n (%).

* Legal context categorisations from Platt and colleagues (2018).^22^ Full criminalisation prohibits all aspects of sex work and selling and buying sex; partial criminalisation criminalises only some aspects; in criminalisation of purchase of sex models, the sale of sex is legal but clients are criminalised; and regulatory models allow the sale of sex in some settings or conditions. Full decriminalisation removes all criminality of sex work while still prohibiting violence and coercion of sex workers.

† Can be in more than one category.

‡ Study categorisations used are derived from the Effective Public Health Practice Project's Quality Assessment Tool for Quantitative Studies.^33^

§ One study evaluated both outreach and static interventions.

Most studies were from North America. 11 (55%) focused on street-based sex workers, and nearly all exclusively studied female sex workers. Eight (40%) of the interventions were multicomponent. 18 (90%) of the interventions (90%) took place in a context where sex work was fully or partly criminalised at the time of study. Interventions were primarily based in static locations, although seven (35%) studies included outreach components. The most common outcomes measured related to drug use and drug harm reduction, sexual risk behaviours, and mental health and wellbeing. No harms associated with the interventions were reported. All studies presented limitations in sampling strategy. Most used convenience or snowball sampling. A few used repeated time-space sampling of mapped sex worker districts to improve systematicity.^26^, ^38^, ^41^, ^42^, ^49^ Four (20%) studies were randomised controlled trials, but all had limitations including non-systematic recruitment strategies,^37^, ^38^, ^40^, ^41^ an absence of information on the randomisation process,^38^, ^40^ and no data on loss to follow-up.^40^ Only one was reported using Consolidated Standards of Reporting Trials guidelines.^37^

**Figure 2:**
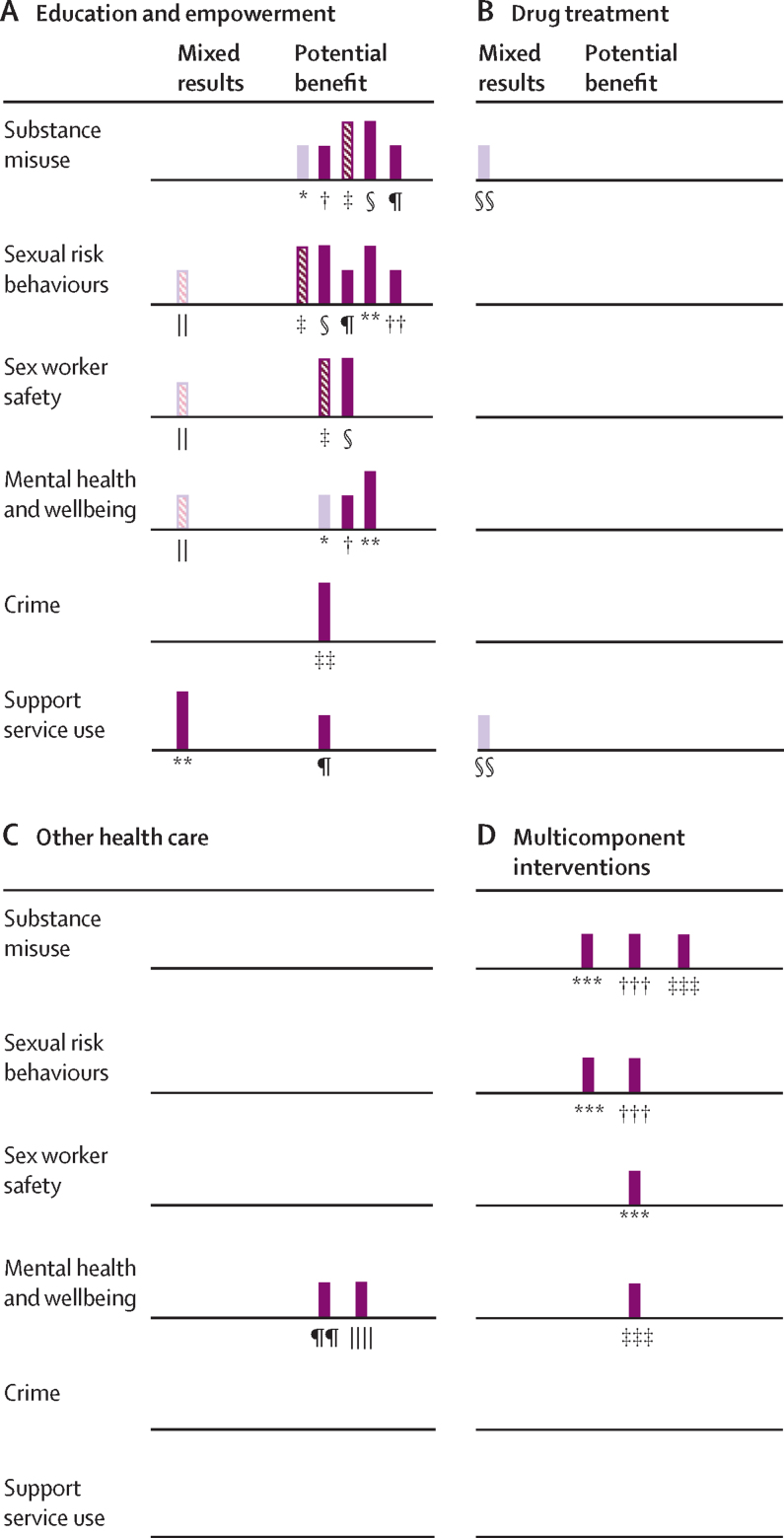
Harvest plot of evidence for interventions to improve health and wider determinants in sex workers by intervention category The harvest plot is a supermatrix showing the direction of effect for outcome categories across different categories of intervention. Each bar represents an intervention and is labelled by a footnote, which can be identified below. Taller bars represent interventions from studies with a moderate-quality assessment; shorter bars are interventions from studies with a low-quality assessment. Darker purple bars are static interventions. Lighter purple bars are outreach. Striped bars are interventions that involved peer design, delivery, or both. Solid bars had no peer involvement. *Murnan et al (2018)^40^—home intervention. †Murnan et al (2018)^40^—office intervention. ‡Surratt and Inciardi (2010)^38^—sex worker-focused intervention. §Surratt and Inciardi (2010)^38^—National Institute on Drug Abuse intervention. ¶Surratt et al (2014)^41^—professional–peer intervention. ||Decker et al (2017).^44^ **Wong et al (2019)^37^—resilience programme. ††Burnette et al (2009).^14^ ‡‡Cigrang et al (2020).^39^ §§Park et al (2020).^43^ ¶¶Ward and Roe-Sepowitz (2009)^9^—prison group intervention. ||||Ward and Roe-Sepowitz (2009)^9^—community group intervention. ***Litchfield et al (2010).^45^ †††Sherman et al (2006).^47^ ‡‡‡Bowser et al (2008).^46^

The harvest plot (figure 2) summarises evidence for effectiveness within each intervention category across different outcomes. A total of 15 interventions from 12 studies could be included within the harvest plot. Nine (60%) of 15 included interventions were focused on education and empowerment and many showed improvements in one or more outcome. Multicomponent interventions showed potential benefit, although only three (15%) interventions were included and all were of low quality. Only a small amount of evidence could be included for drug treatment and other health-care interventions; however, drug treatment was a central component of one of the multicomponent interventions (Litchfield and colleagues [2010]).^45^ No studies based on sexual and reproductive health care could be included. With the exception of Decker and colleagues (2017),^44^ which only included a peer-design element, all other interventions involving peers included both a design and delivery element and showed potential benefit. Three outreach interventions were included, of which one showed potential benefits across outcomes,^40^ and two showed mixed results.^43^, ^44^

We analysed the number of positive outcomes (potential benefit) to the number of total outcomes reported per intervention category using the binomial exact calculation and binomial probability test (table 3). Education and empowerment and multicomponent interventions showed a greater proportion of positive outcomes than would have been expected by chance, suggesting their potential effectiveness, whereas the little evidence for drug treatment and other health-care interventions precludes clear insight.

**Table 3:** Effectiveness of intervention categories across different outcome measures

	**Number of potentially beneficial outcomes of total outcomes**	**95% CI of the proportion of beneficial outcomes**	**p value**
Education and empowerment	17 (80%) of 21	58·1–94·6	0·007
Drug treatment	0 (0%) of 2	0–84·2	0·50
Other health care	0 (0%) of 2	0–84·2	0·50
Multicomponent interventions	7 (100%) of 7	59·0–100	0·016

For each intervention category, a p value was calculated using the binomial probability test to determine the chance that the true proportion of potentially beneficial outcomes of total outcomes was 0·50. Accompanying exact 95% binomial CIs are also displayed.

### Education and empowerment

Seven interventions (six single component,^37^, ^38^, ^39^, ^40^, ^41^, ^44^ and one multicomponent^47^) focused on education and empowerment, and four multicomponent interventions had a small educational component, but did not detail what was provided.^45^, ^46^, ^51^, ^52^ Of the seven, three focused on street-based sex workers,^38^, ^41^, ^47^ one on street-based and indoor-based sex workers,^44^ and in the other three the authors did not state the sex worker population that the intervention was targeting.^37^, ^39^, ^40^ Three were of moderate quality,^37^, ^38^, ^39^ and four were of weak quality.^40^, ^41^, ^44^, ^47^

A few studies used health behaviour models that recognise structural and environmental vulnerabilities contributing to HIV and sexual health risk.^37^, ^47^ Structural determinants were addressed through enhancing sex worker self-efficacy and condom negotiation skills,^37^, ^47^ as well as teaching strategies to minimise risk of violence.^38^, ^44^ Several used psychological therapies—five were individually administered,^37^, ^38^, ^39^, ^41^, ^44^ and one used family therapy between mothers who were sex workers and their children.^40^ Two were developed and delivered in collaboration with peer sex workers.^38^, ^41^ Key study outcomes for these interventions related to sexual risk behaviours,^37^, ^38^, ^41^, ^47^ drug use and drug harm reduction,^38^, ^41^, ^47^ mental health and wellbeing,^37^, ^40^, ^44^ use of other health-care and support services,^37^, ^41^ and criminal activity.^39^

All interventions showed a level of effectiveness, but most only measured outcomes at 3 months after intervention.^37^, ^44^, ^47^ A brief intervention that provided information on strategies to improve sex worker safety and reduce the risk of violence affected safety behaviours and use of relevant support programmes 12 weeks later.^44^ A six-session resilience-promoting programme showed improvements in resilience, self-esteem, and condom use 3 months later.^37^ A 12-session family therapy programme showed greater reductions in drug use and depressive symptoms than a psychoeducational programme with only sex workers.^40^ One programme helped sex workers to develop negotiation skills with different sexual partner types alongside teaching jewellery-making skills.^47^ 3 months post-intervention, there were reductions in transactional and total sex partners, as well as injection and non-injection drug use. In another intervention, female sex workers in prison were provided two brief motivational interviews to help identify and problem solve their greatest concerns for post-release.^39^ This intervention led to a reduction in the number of arrests in the 12-month period after release.

Two studies including peer sex workers in the development and delivery of an intervention showed mixed results.^38^, ^41^ Both were randomised controlled trials with interventions showing similarly positive outcomes to control groups, which were high quality. One found that a strengths-based programme did not show additional effectiveness when incorporating a peer facilitator over a case manager alone.^38^ The other study showed that a sex worker-focused HIV risk education programme, developed and delivered in collaboration with sex workers, led to a significantly greater reduction in unprotected oral sex and episodes of sexual violence than the US National Institute on Drug Abuse standard intervention at 6-month follow-up. However, other HIV risk outcomes were similar to the standard intervention.^41^

### Drug treatment

Drug treatment was provided by four interventions (two single component^14^, ^43^ and two multicomponent^45^, ^48^). Three specifically targeted sex worker populations—a drug treatment clinic for street-based female sex workers,^45^ a one-off harm reduction intervention for street-based female sex workers,^43^ and a one day per week clinic offering an array of primary care and harm reduction services to an unspecified sex worker population.^48^ The fourth study compared outcomes between sex workers (no subpopulation identified) and non-sex workers using US-Government-funded drug treatment programmes across 71 facilities.^14^ Three interventions were at static locations,^14^, ^45^, ^48^ and all studies were low quality.

The primary care clinic studied by Stewart and colleagues^48^ found that 31 (62%) of 50 women seen had opioids in their urine. Of these, nine (29%) of 31 started opioid substitution therapy (OST) and three (10%) were already in OST programmes. In the harm reduction intervention, women were given naloxone, harm reduction advice, and self-administered tests for detecting the presence of fentanyl in drugs.^43^ Fentanyl has a higher risk of overdose and death compared with heroin. 1 month after intervention, opioid and injection drug use, as well as solitary drug use, had reduced. However, fentanyl detection in drugs did not lead to changes in harm reduction behaviours for most people. The other two studies on drug treatment found a significant decrease in drug use at the end of drug programmes (one focused on heroin,^45^ the other included various drugs^14^), and a reduction in the number of women still engaging in sex work.^14^, ^45^ Burnette and colleagues^14^ found that those still involved in sex work were doing significantly less sex work than they had before. Both interventions provided physical and mental health services alongside OST, which led to improvements in mental health and wellbeing. Burnette and colleagues^14^ found higher use of mental health services was associated with increased probability of cessation of sex work at follow-up, which in turn was associated with lower drug use, higher abstinence rates, and fewer mental health symptoms.^14^

### Sexual and reproductive health care

Two interventions provided STI screening, STI treatment, and HIV pre-exposure prophylaxis through sexual health outreach clinics in brothels,^51^ and a 1 day per week, multicomponent primary care intervention for an unspecified sex worker population.^48^ Two multicomponent welfare services for street-based sex workers provided free condoms and lubricants.^26^, ^42^, ^50^ One study described the management of a syphilis outbreak in street-based sex workers in east London.^53^ Through partnership with a charity providing outreach to sex workers, women with suspected syphilis were invited to the charity's drop-in centre, from where they were driven to a nearby genitourinary medicine clinic. Reproductive health-care service components included pregnancy testing,^48^ contraceptive prescriptions and advice,^45^, ^52^ and post-coital contraception.^51^ No details of service provision, uptake, or acceptability were provided and no study focused on reproductive health care.

Studies were observational and low quality. Two provided information on STI treatment.^51^, ^53^ Sturrock and colleagues^51^ invited sex workers with positive results back for treatment. 42 (62%) of 68 participants returned for their results and seven (17%) of 42 returning sex workers received treatment. In the syphilis outbreak, epidemiological treatment (ie, treatment based on probable exposure) was provided to all sex workers.^53^ Most individuals declined intramuscular penicillin—the best available treatment—and many instead chose oral antibiotics, which are a suboptimal alternative. 13 (93%) of 14 sex workers were followed up.

### Other health care

Other health-care interventions included a trauma-based psychoeducational therapy group for street-based sex workers,^9^ a vaccination programme for multiple sex worker populations,^49^ a multicomponent clinic focused on women's health for street-based female sex workers,^52^ and multicomponent primary care clinics in the proximity of welfare drop-in centres.^26^, ^48^ The vaccination programme was a nationally run programme in the Netherlands. The programme provided free hepatitis B vaccinations to sex workers through local community health services, working alongside existing sex worker outreach services, and by community health-service staff periodically visiting various sex work locations, including brothels and streets over several years. All other interventions were at static locations, with the therapy group provided at both a community-based residential centre and a moderate-security prison. All studies were low quality. Several interventions offered referral to other health or social services, but no study gave information on the uptake of this offer.^26^, ^38^, ^40^, ^42^, ^50^, ^52^

The psychotherapy group participants' trauma scores decreased significantly in six of ten parameters at the end of the 12-week intervention.^9^ The decrease was more profound in the prison group than in the residential centre, possibly as their baseline trauma scores were higher. Baars and colleagues^49^ provided evidence of the effectiveness of the Netherlands' hepatitis B vaccination programme. Through a cross-sectional survey of 259 sex workers working in various settings across three cities, they found that 2 years after programme initiation, 205 (79%) of 259 were aware of the programme and 163 (63%) of 257 had received at least one dose—134 (82%) of 163 through the programme. Of those who started the vaccine programme, 74 (79%) of 94 received all three vaccinations. Those who had been vaccinated were more likely to have worked in an area for longer and 75% reported receiving their first vaccination at an outreach location. Wong evaluated a well-women clinic's cervical cancer screening intervention for street-based female sex workers in Hong Kong.^52^ 208 (88%) of 236 tested women returned for their smear results, and 13 (45%) of 29 women with atypical smear results were uncontactable. Nine (31%) of the 29 with atypical results were given referral letters to attend a gynaecologist, but it is not known whether they were seen.

### Welfare

All interventions that addressed welfare were multicomponent and focused on meeting basic needs through providing food and drink, washing facilities, clothing, and a safe space.^26^, ^42^, ^46^, ^50^ The intervention by Sherman and colleagues^47^ was the only exception which, alongside teaching better condom negotiation skills, taught female sex workers jewellery-making skills over six sessions. These women then had the opportunity to sell their handmade items at a stand within a hospital. The intervention was designed to address structural determinants preventing these women earning a sustainable, alternative income. 3 months after completion, there were significant reductions in transactional and total sex partners, as well as injection and non-injection drug use.^47^ Women who earned more money through market sales had a significantly decreased number of transactional sex partners at follow-up. All welfare interventions were oriented towards street-based sex workers and studies were of low quality.

Three studies focused on two linked interventions in Vancouver, BC, Canada.^26^, ^42^, ^50^ The interventions, both designed for female sex workers, were the Women's Information Safe Haven (WISH) drop-in centre and a peer-led, van-based outreach programme called the Mobile Access Project (MAP). Those with greater numbers of clients and working in isolated areas were more likely to use the MAP van,^42^ reflecting the outreach approach used. The studies showed that both services were associated with accessing other health services—inpatient addiction services for the MAP van,^42^ and sexual and reproductive services for WISH.^26^ However, the temporality of both relationships is unclear. Both the MAP van and WISH were less likely to be used by younger sex workers compared with older sex workers. Of those who used the MAP van, 94% felt safer when the van was present, 16% recalled a time it had prevented physical assault, and 10% a time it had prevented sexual assault.

## Discussion

We identified 20 studies, with intervention components divided into education and empowerment, drug treatment, sexual and reproductive health care, other health care, and welfare. 12 interventions were single component and eight were multicomponent. Considering the diversity of sex worker populations and their corresponding needs, this was a very small number of studies. There was promising evidence for interventions that focused on education and empowerment and those that were multicomponent. Sherman and colleagues'^47^ jewellery skills and sexual negotiation strategy workshops were particularly innovative as a multicomponent intervention combining empowerment and a focus on the structural determinants of health. Evidence across studies also showed that designing and delivering interventions alongside sex workers was effective. Importantly, only six interventions used co-design or co-delivery. The harvest plot provided unclear results as to the effectiveness of outreach. However, two interventions that involved outreach, but could not be included in the plot because they were cross-sectional studies and did not follow up participants, showed evidence of possible benefit. Both the Netherlands' hepatitis B vaccination programme^49^ and the management of a syphilis outbreak in east London^53^ relied on collaboration with existing outreach services and showed good uptake and retention. Few interventions incorporated reproductive health care,^48^ and there was no evidence for interventions treating chronic diseases. One intervention provided cervical cancer screening, but many people with atypical results could not be contacted and informed, and it is unclear whether those who were contacted were followed up.^52^

Previous systematic reviews analysing health interventions for sex workers in high-income countries underscored the need for flexible services which are non-judgemental, built on respect and trust, trauma-informed, and targeted at specific sex worker needs.^10^, ^29^, ^30^ Our Review also found outreach might be important in ensuring high levels of engagement in some contexts. However, outreach was not always enough to ensure continuity of care. Similar to other systematic reviews,^10^, ^29^ we found many studies highlighted low levels of follow-up. Three studies were exceptions to this. One involved OST,^45^ possibly showing the perceived value of this intervention. The other two,^49^, ^53^ as discussed earlier, collaborated with established outreach services, which might have improved trust and provided a channel by which to follow up individuals.

To the best of our knowledge, this Review is the first comprehensive overview of evidence on sex worker interventions aiming to improve health and wider determinants of health outcomes in high-income countries. Academic databases and grey literature were searched, and both academic experts and people with lived experience of sex work were contacted to ensure we identified all relevant literature. Importantly, we have included authors with lived experience, and authors who have worked with and continue to work with sex workers, from the study's inception—to develop the search strategy, ensuring relevant grey literature channels were searched and experts in the field contacted, and to ensure findings were relevant, correctly interpreted, and presented with appropriate language and without stigma.

This Review has some limitations. Where stated, the majority of interventions were either primarily or exclusively targeted at street-based sex workers,^26^, ^38^, ^41^, ^42^, ^43^, ^45^, ^46^, ^47^, ^50^, ^52^, ^53^ probably because they are more easily identified by service providers and researchers; are more exposed to structural determinants such as homelessness, poverty, and violence;^18^, ^54^, ^55^ and typically have worse health outcomes.^56^ Therefore, generalisability of this Review's findings to other sex worker populations is limited. People engaged in street-based sex work often have a range of different health and social issues, including homelessness,^4^, ^18^ drug use,^10^, ^14^ and history of imprisonment,^10^ emphasising the need for a wider inclusion health approach to service provision and research that addresses multiple, overlapping risk factors and vulnerabilities.^28^ We reviewed English language studies since 2005 as a pragmatic choice and because an initial scoping search suggested most studies relevant to this Review met these criteria. We did not include qualitative studies that might provide insight into differences in results between studies. Outcomes were highly heterogeneous, often self-reported, and might not be the outcomes that are important for all sex workers. The development of a core outcome set in collaboration with sex workers would help future researchers to ensure that outcomes measured are relevant.^57^ Methods used by the included studies also represent an important limitation, with only three studies^37^, ^38^, ^39^ rated moderate in our quality assessment, and all other studies rated weak (table 2). One common reason for low quality was study design—the most common design was a single group, pre-post cohort study (often referred to as quasi-experimental studies). Additionally, due to the nature of recruiting marginalised populations, all studies presented limitations in sampling strategy and most used either convenience or snowball sampling. Finally, the complexity and dynamic nature of the legal sex working context in which the interventions took place could not be accounted for in the Review's findings and is likely to be an explanatory factor for study heterogeneity.

There is scarce investment both in services and research, particularly for sex workers who are not street based. However, a range of interventions are likely to be effective. Services should be developed and delivered in collaboration with sex workers. Interventions that are focused on education and empowerment or those that are multicomponent are likely to be effective, and an outreach or drop-in component could be of benefit in some contexts.^58^ Future interventions should incorporate components related to chronic diseases given they are an important contributor to sex worker mortality.^58^ Within the identified studies, almost all interventions were designed exclusively for female sex workers—the only exceptions being two that included transgender women sex workers,^26^, ^42^ and one that included male sex workers.^51^ Sex worker services and future research should take a gender-sensitive and inclusive approach. Several studies highlighted that sex workers who were new to working in an area were less likely to access services than those who had been working in an area for longer.^26^, ^42^, ^49^ Effective information dissemination and outreach could help ensure accessibility. Crucially, repressive policing practices and the criminalisation of sex work have already been shown to adversely affect access to health and social services and sex worker health outcomes.^22^ Therefore, the effectiveness of any service will always be restricted in settings where sex work is criminalised.

## Declaration of interests

SAL and LCP are Pathway Fellows. Pathway is a charity that provides health care to homeless and inclusion health patients, including sex workers. All other authors declare no competing interests.
