# Supplementary material for: Interventions to improve health and the determinants of health among sex workers in high-income countries: a systematic review
Source: Lancet Public Health. 2022 Nov 2;8(2):e141–54. doi: 10.1016/S2468-2667(22)00252-3 (PMC10564624; doi:10.1016/S2468-2667(22)00252-3)

THE LANCET
Public Health

Supplementary appendix

This appendix formed part of the original submission and has been peer reviewed. We post it as supplied by the authors.

Supplement to: Johnson L, Potter LC, Beeching H, et al. Interventions to improve health and the determinants of health among sex workers in high-income countries: a systematic review. *Lancet Public Health* 2022; published online Nov 2. https://doi.org/10.1016/S2468-2667(22)00252-3.

Supplementary appendices

Appendix A: Details of search strategy……………………………………………………………………………………………2

Appendix B: A map of the number of studies in each intervention component category within each country……….4

Appendix A: Details of search strategy

Language: English

Date of publication: 1st Jan 2005-16th December 2021

Search was conducted in each database using both Keywords and Subject Headings for each term, with AND between each term.

For both keywords and subject headings

1=sex worker terms

2= health intervention

Example (1 keywords OR 1 headings) AND (2 keywords OR 2 headings)

Note: Cochrane and Web of Science databases were searched without the use of keywords because of the nature of the databases.

Keywords: Combine 1, 2 with AND

1. **Sex workers**

   sex work* OR prostitut* OR street walker* OR escort* OR rent boy* OR sell sex OR sold sex OR selling sex OR exchanged sex OR sex trade OR commercial sex OR sex industry OR transactional sex OR sexual favour OR bar hostess OR red light district

2. **Health or social care Interventions**

   clinic* OR treatment OR outreach OR needle exchange progra* OR hospital* OR health care OR health service* OR psychologist* OR psychiatrist* OR counselling OR casework* OR hous* OR accommodation OR shelter* OR social services OR social welfare OR social security OR primary care OR GP OR general practi* OR family doctor* OR dentist* OR dent* OR test* OR treat* OR screen* OR assess* OR outreach OR specialist service OR social care* OR voluntary sector* OR voluntary service* OR community care* OR social service OR safeguarding OR harm reduction OR methadone OR buprenorphine OR opioid substitution OR naloxone OR hospitalisat* OR emergenc* OR prescription OR public health OR prevent* OR intervent*

Subject Headings: Combine 1 AND 2

	**1) Sex work terms**	**2)Intervention terms**
**Embase Via Ovid**	Exp Prostitution	Exp early intervention/ OR Exp community program/ OR Exp community care/ OR Exp public health/ OR Exp health promotion/ OR exp drug program/ OR Exp support group/ OR Exp health service/ OR Exp hospital service/ OR Exp health care utilization/ OR Exp medical service/ OR Exp mental health/ OR Exp drug dependence treatment/ OR Exp drug rehabilitation program/ OR Exp Primary Healthcare/ OR Exp General Practitioners/

**PsychInfo**	Exp Prostitution/ OR Exp Sex work/ OR Exp transactional sex	Exp Intervention/ OR Exp early intervention/ OR Exp support groups/ OR Exp health care utilization/ OR Exp mental health services/ OR Exp hospitalization/ OR Exp hospital programs/ OR Exp health care delivery/ OR Exp health policy/ OR Exp health promotion/ OR Exp community services/ OR Exp Primary Health Care/ OR Exp Family Physicians/ OR Exp General Practitioners/ OR Exp Physicians/ OR Exp harm reduction/ OR Exp Methadone/ OR Exp Buprenorphine/ OR Exp Substance use treatment/ OR Exp public health/ OR Exp social casework/
**Cochrane Library**	n/a	n/a
**MEDLINE**	Exp Sex workers	Exp intervention/ OR Exp Early Intervention (education)/ OR Exp Crisis intervention/ OR Exp Community Mental Health services/ OR Exp community services/ OR Exp Health promotion/ OR Exp Harm reduction/ OR Exp drug dependence treatment/ OR Exp drug rehabilitation program/ Exp Community Health services/ OR Exp Health services/ OR Exp health care utilization/ OR Exp Hospitalization/ OR Exp Social Work/ OR Exp Public Health/ OR Exp Self-help groups/ OR Exp mental health services/ OR Exp Primary Healthcare/ OR Exp Family Physicians/ OR Exp General Practitioners/
**CINAHL**	Exp Prostitution	Exp Early Intervention/ OR Exp Support group/ OR Exp health services needs and demand/ OR Exp Health Services Accessibility/ OR Exp Health care delivery/ OR Exp Community Mental Health services/ OR MM community programs/ OR MM Primary Health care/ OR Exp Substance Use Treatment / OR Exp Substance use rehabilitation programs / Exp Hospitalization/ Or MM hospital programs/ OR Exp Social Work/ OR Exp Support Groups/ OR Exp Health Promotion/ OR Exp Public health
**Web of Science**	n/a	n/a

** Nursing intervention classification system

Appendix B: A map of the number of studies in each intervention category within each country

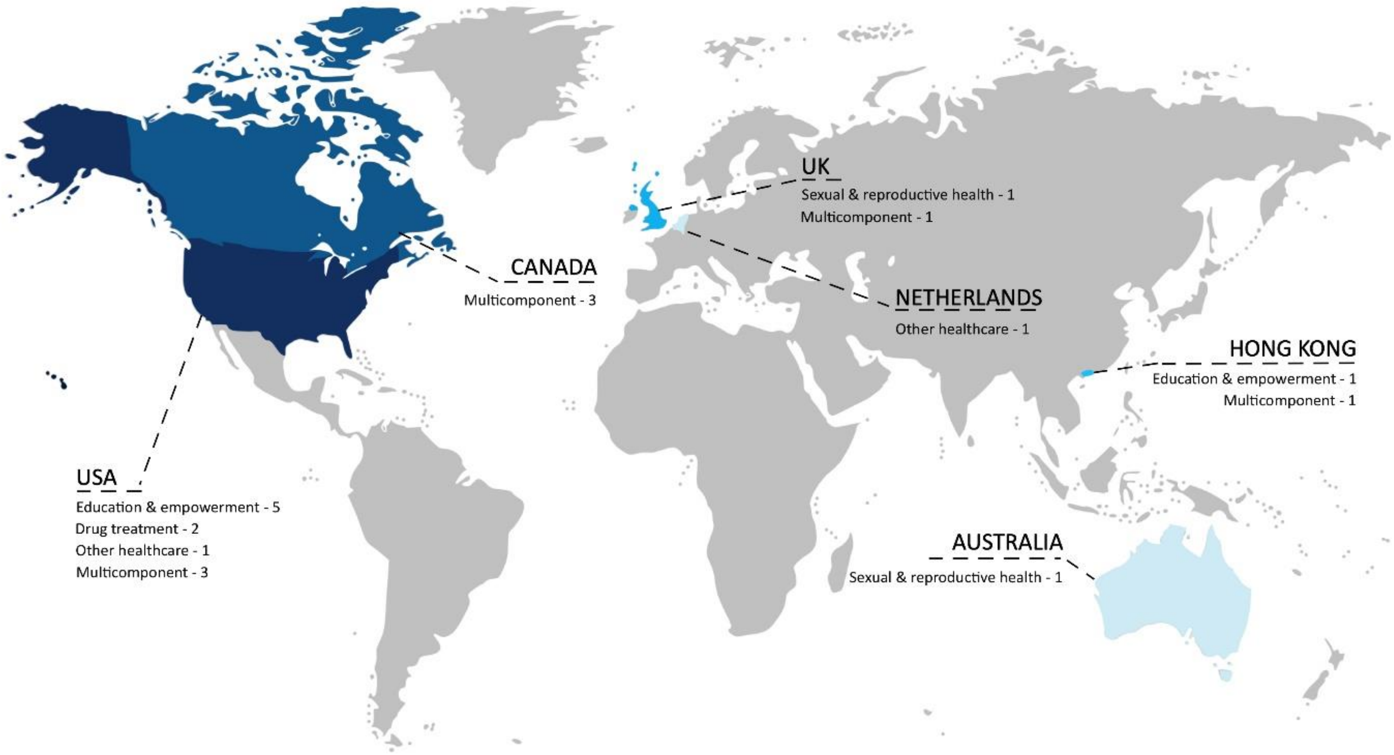

Supplement: Supplementary appendix [file mmc1.pdf]
